# Effects of *FTO* and *PPARγ* variants on intrauterine growth restriction in a Brazilian birth cohort

**DOI:** 10.1590/1414-431X202010465

**Published:** 2021-03-04

**Authors:** M.R. Barbieri, A.M. Fontes, M.A. Barbieri, M.C.P. Saraiva, V.M.F. Simões, A.A.M. da Silva, K.J. Abraham, H. Bettiol

**Affiliations:** 1 Departamento de Puericultura e Pediatria, Faculdade de Medicina de Ribeirão Preto, Universidade de São Paulo, Ribeirão Preto, SP, Brasil; 2 Departamento de Genética, Faculdade de Medicina de Ribeirão Preto, Universidade de São Paulo, Ribeirão Preto, SP, Brasil; 3 Departamento de Clínica Infantil, Faculdade de Odontologia de Ribeirão Preto, Universidade de São Paulo, Ribeirão Preto, SP, Brasil; 4 Departamento de Saúde Pública, Centro de Ciências da Saúde, Universidade Federal do Maranhão, São Luís, MA, Brasil; 5 Departamento de Economia, Faculdade de Economia, Administração e Contabilidade de Ribeirão Preto, Universidade de São Paulo, Ribeirão Preto, SP, Brasil

**Keywords:** IUGR, FTO, PPARγ, SNPs, Cohort study

## Abstract

Intrauterine growth restriction (IUGR) is related to a higher risk of neonatal mortality, minor cognitive deficit, metabolic syndrome, and cardiovascular disease in adulthood. In previous studies, genetic variants in the *FTO* (fat mass and obesity-associated) and *PPARγ* (peroxisome proliferator-activated receptor-gamma) genes have been associated with metabolic disease, body mass index, and obesity among other outcomes. We studied the association of selected *FTO* (rs1421085, rs55682395, rs17817449, rs8043757, rs9926289, and rs9939609) and *PPARγ* (rs10865710, rs17036263, rs35206526, rs1801282, rs28763894, rs41516544, rs62243567, rs3856806, and rs1805151) single-nucleotide polymorphisms (SNPs) with IUGR, through a case-control study in a cohort of live births that occurred from June 1978 to May 1979 in a Brazilian city. We selected 280 IUGR cases and 256 controls for analysis. Logistic regression was used to jointly analyze the SNPs as well as factors such as maternal smoking, age, and schooling. We found that the *PPARγ* rs41516544 increased the risk of IUGR for male offspring (OR 27.83, 95%CI 3.65-212.32) as well as for female offspring (OR=8.94, 95%CI: 1.96-40.88). The *FTO* rs9939609 TA genotype resulted in a reduced susceptibility to IUGR for male offspring only (OR=0.47, 95%CI: 0.26-0.86). In conclusion, we demonstrated that *PPARγ* SNP had a positive effect and *FTO* SNP had a negative effect on IUGR occurrence, and these effects were gender-specific.

## Introduction

Intrauterine growth restriction (IUGR) is known to be related to a higher risk of neonatal mortality, minor cognitive deficits, school problems, metabolic syndrome (MetS), and cardiovascular disease in adulthood (1–4).

Although the consequences of IUGR have been widely reported, causal explanations are still unclear. In addition to suggestions about the importance of maternal, fetal, placental, and genetic factors or any combination thereof (5,6), other hypotheses have been proposed, among them: 1) the Barker hypothesis, which suggests that the maternal uterine environment in pregnancy has long-term effects in adulthood resulting in increased metabolic disease and diabetes risk (1,7); 2) the fetal insulin hypothesis, which proposes that the insulin secreted by the fetal pancreas in response to the maternal glucose concentration affects both newborn size and predisposition to adult diseases (8); and 3) the genetic factors hypothesis, which is supported by the observation that IUGR tends to cluster in families and recur in successive pregnancies (9,10). In this regard, a number of single-nucleotide polymorphisms (SNPs) in genes involved in the efficiency of the metabolism of specific macronutrients may have a relevant role for adult MetS in different populations (11–13).

Several SNPs of the human *FTO* gene (fat mass and obesity-associated, NM_001080432) have been associated with body mass index (BMI) (13,14) and susceptibility to type 2 diabetes mellitus (T2DM) and obesity (15,16). In the same way, several allelic variants of the *PPARγ* gene (peroxisome proliferator-activated receptor-gamma) have been associated with MetS and T2DM (17–20). A meta-analysis suggests that *FTO* may represent a low-penetrance susceptible gene for obesity that may vary according to ethnic composition of the population (21). Considering that IUGR is associated with MetS in adulthood and allele variants of the *FTO* and *PPARγ* genes are associated with obesity and MetS later in life, it is plausible to hypothesize that these allele variants may also have a role in intrauterine growth, with potential effects on obesity and metabolic disorders later in life.

Few studies have investigated the possible effect of *FTO* and *PPARγ* SNPs on fetal growth and the results are controversial. The rs9939609 A:A *FTO* genotype was associated with faster biparietal diameter and head circumference growth velocities between 11 and 34 weeks of gestation than the T:T *FTO* genotype (22). The *FTO* rs9939609 A:A genotype has an influence on fetal growth restriction after 28 weeks of gestation (22). The *FTO* gene has also been studied in relation to the small for gestational age (SGA) condition (13), which is closely related to IUGR (23). Although there are studies that were unable to find association between fetal growth and *PPARγ* SNPs (24), others have shown a reduced expression of *PPARγ* in placentas of SGA children, maybe related to higher DNA methylation (25), which is more prone to arise after the first trimester of gestation (26). However, if methylation is a cause or a consequence of SGA is still unknown.

Thus, in view of these controversies, this paper examines the frequency of selected *FTO* and *PPARγ* SNPs with regard to their association with IUGR in a Brazilian cohort, while taking into account additional confounding factors also potentially related to IUGR.

## Material and Methods

### Subjects

This was an unmatched case-control study nested in a prospective cohort study started in 1978/79 in Ribeirão Preto, involving children born in hospitals to mothers residing in the municipality (23). The birth cohort consists of a total of 9067 children born in eight medical facilities in Ribeirão Preto from June 1978 to May 1979, corresponding to 98% of live infants. Only 3.5% of the infants were excluded due to refusal to participate or early discharge. After the exclusion of children whose families did not reside in the municipality (2094) and of twins (146), 6827 individuals of the initial sample were available for the study.

The sample size was calculated considering a 95% confidence level, a power of 80%, and an odds ratio of 2.1 estimated *a priori*. Considering a frequency of 43% for the rs9939609 A allele (14) and a ratio of one control to each case, the minimum number of participants in the analysis was calculated in 232, i.e., 116 cases and 116 controls. Cases and controls for the present study were selected from participants in the follow-up of 2002/3 (n=2063) when blood samples were obtained. All individuals defined as having IUGR at birth were included as cases (n= 280). IUGR was defined by the birth weight ratio (BWR), calculated by the ratio between the child's weight and the median weight for gestational age according to the William's curve (27), and growth restriction was defined by a BWR ≤0.85. An additional 276 non-IUGR children from the follow-up were selected at random. Blood samples of all 556 participants were available and were used for genotyping.

The participants were stratified according to gender, as females are more likely to develop IUGR (28), with 253 being males (45.7%) and 303 being females (54.3%). The statistical analysis was performed separately for each sex.

In order to study the effects of genotypes on IUGR, the confounding effects of the following covariates at birth were included: maternal education in years of schooling (<9 and ≥9), maternal smoking during pregnancy (yes, if smoked at least one cigarette per day), and maternal age (<20, 20 to 34, ≥35 years). The study was reviewed and approved by the Research Ethics Committee of the Ribeirão Preto Medical School, University of São Paulo (Process No. 7609/99), and a biological bank of all blood samples was approved later (Process No. 10761/2008).

### Polymerase chain reaction

Genomic DNA was obtained from 40 mL of a peripheral blood sample collected from each participant. The DNA extraction was performed using the FlexiGene DNA (250) Kit (Qiagen, USA), according to the manufacturer's recommendations. The extracted genomic DNAs were quantified using NanoDrop equipment (ThermoScientific, USA), according to the manufacturer's instructions.

SNP analysis was performed by HRM (high-resolution melting) technique. First, the regions of interest of the *FTO* and PPARγ genes were amplified by PCR (polymerase chain reaction). To identify the *FTO* SNPs, the following primer pairs were used: 1) forward 5′-CCATGAGCCAGATTTTCCAT-3′ and reverse 5′-TGGAGGTCAGCACAGAGG-3′ (rs1421085 and rs55682395); 2) forward 5′-CGGTGAAGAGGAGGAGATTG-3′ and reverse 5′-CCCAGTTTCTCCAAGGAAGC-3′ (rs17817449 and rs8043757); and 3) forward 5′-AACTGGCTCTTGAATGAAATAGG-3′ and reverse 5′-TGCTCTCCCACTCCATTTCT-3′ (rs9926289 and rs9939609). To identify the *PPARγ* SNPs, the following primer pairs were used: 1) forward 5′-TCATGTAGGTAAGACTGTGTAG-3′ and reverse 5′-AACCAGAGAGCAGATCATC-3′ (rs10865710 and rs17036263); 2) forward 5′-AACGGATTGATCTTTTGCTAG-3′ and reverse 5′-TGGAAGACAAACTACAAGAGC-3′ (rs35206526 and rs1801282); and 3) forward 5′-AGGTTTGCTGAATGTGAAG-3′ and reverse 5′-GGAAGAAGGGAAATGTTGG-3′ (rs28763894, rs41516544, rs62243567, rs3856806, and rs1805151). The equipment used for amplification of the corresponding fragments was the Gene Amp PCR System 9700 (Applied Biosystems, USA).

Then, SNPs analysis by HRM on the DNA samples of the selected individuals was performed on the ViiA7 Real Time PCR System (ThermoFisher Scientific), according to the manufacturer's instructions.

### DNA sequencing

Sequencing of samples to confirm the presence or absence of SNPs in the candidate genes was performed using the ABI Prism Big Dye Terminator Cycle Sequencing kit (Applied Biosystems), according to the manufacturer's instructions. Then, the samples were electrophoresed on the ABI Prism 3130 automated sequencer (Applied Biosystems). Some samples were used as control variants after PCR, but during the analysis by HRM some samples that differed from the control variants were also sequenced to identify and confirm the SNPs.

### Statistical analysis

First, for quality control, all polymorphisms were tested for Hardy-Weinberg equilibrium (HWE) using the Hardy-Weinberg Library in the R programming language. The criterion used to discard a given polymorphism was based on a violation of HWE with a P-value less than 0.001 using only the controls (29). With this criterion, all polymorphisms with fixed genotypes were automatically excluded.

In order to decide which covariates to include in the adjusted analysis, we treated IUGR as a categorical factor and computed the unadjusted P-values between IUGR and other risk factors. For each risk factor, a chi-squared test was performed to assess the strength of association with growth restriction.

For each gender group, the association of the SNPs and IUGR was assessed by multivariate logistic regression separately. The partial odds ratio (OR) for each covariate was calculated and 95% confidence intervals for all ORs were obtained. Statistical significance was based on a 5% threshold. All genotypes were treated without reference to a specific genetic model such as dominance of one allele or an additive model. In the final statistical analysis, only individuals with no missing covariates were retained.

## Results

Among the 556 participants, we determined the genotype frequencies of 14 SNPs and one indel; six of them were in the *FTO* gene and eight of them and one indel in the *PPARγ* gene. A complete description of the polymorphisms in the *FTO* and *PPARγ* genes is shown in Table 1. Our results showed that five of the markers were not polymorphic in this population. As seen, one SNP in the *FTO* gene (rs55682395), three SNPs and one indel in the *PPARγ* gene (rs28763894, rs62243567, rs1805151, and rs35206526) were fixed and showed no variation across different individuals.

**Table 1:** Genotype frequencies and classification of single-nucleotide polymorphisms (SNPs) of the *FTO* (fat mass and obesity-associated) and *PPARγ* (peroxisome proliferator-activated receptor-gamma) genes.

Polymorphisms (GRCH38.p12)	Genotype	Participants (n=556) n (%)	Classification
*FTO*			
rs1421085 T>C	T/T	229 (41.2)	Intron variant
chr16: 53767042	T/C	253 (45.5)	
	C/C	74 (13.3)	
rs55682395 C>T	C/C	556 (100)	Intron variant
chr16: 53767031	C/T	0 (0.0)	
	T/T	0 (0.0)	
rs17817449 T>G	T/T	287 (51.6)	Intron variant
chr16: 53779455	T/G	269 (48.4)	
	G/G	0 (0.0)	
rs8043757 A>T	A/A	277 (49.8)	Intron variant
chr16: 53779538	A/T	269 (48.4)	
	T/T	10 (1.8)	
rs9926289 G>A	G/G	205 (36.9)	Intron variant
chr16: 53786591	G/A	247 (44.4)	
	A/A	104 (18.7)	
rs9939609 T>A	T/T	205 (36.9)	Intron variant
chr16: 53786615	T/A	247 (44.4)	
	A/A	104 (18.7)	
*PPARγ*			
rs10865710 C>G	C/C	352 (63.3)	Intron variant
chr 3: 12311699	C/G	204 (36.7)	
	G/G	0 (0.0)	
rs17036263 A>G	A/A	542 (97.5)	Intron variant
chr 3: 12311776	A/G	14 (2.5)	
	G/G	0 (0.0)	
rs35206526 TAGA>-	TAGA/TAGA	556 (100)	Intron variant indel
chr 3: 12351542	TAGA/-	0 (0.0)	
rs1801282 C>G	C/C	479 (86.2)	Missense variant
chr 3: 12351626	C/G	77 (13.8)	p.Pro12Ala
	G/G	0 (0.0)	
rs28763894 A>G	A/A	556 (100)	Synonymous variant
chr 3: 12433941	A/G	0 (0.0)	p.Gln410=
	G/G	0 (0.0)	
rs41516544 A>G	A/A	516 (92.8)	Synonymous variant
chr 3: 12433998	A/G	40 (7.2)	p.Ser429=
	G/G	0 (0.0)	
rs62243567 A>C	C/C	556 (100)	Missense variant
chr 3: 12434056	A/C	0 (0.0)	p.His449Asn
	A/A	0 (0.0)	
rs3856806 C>T	C/C	424 (76.3)	Synonymous variant
chr 3: 12434058	C/T	132 (23.7)	p.His449=
	T/T	0 (0.0)	
rs1805151 C>T	C/C	556 (100)	Synonymous variant
chr 3: 12434068	C/T	0 (0.0)	p.Leu453=
	T/T	0 (0.0)	

The genotype and allele frequencies for the ten polymorphic markers from the *FTO* and *PPARγ* genes separated by IUGR and non-IUGR groups are listed in Table 2. Higher differences in genotype and allele distributions between IUGR and non-IUGR groups were observed for the heterozygous genotypes AG and CT of *PPARγ* SNPs, rs41516544 and rs3856806. For the *FTO* gene, three markers were in HWE (rs1421085, rs9926289, and rs9939609), and for the *PPARγ* gene, there were four SNPs (rs17036263, rs1801282, rs41516544, and rs3856806).

**Table 2:** Genotype and allele frequencies of the polymorphic single-nucleotide polymorphisms (SNPs) from *FTO* (fat mass and obesity-associated) and *PPARγ* (peroxisome proliferator-activated receptor-gamma) genes in intrauterine growth restriction (IUGR) and control groups.

Polymorphisms	Genotype frequency			Allele frequency			HWE P-value
	Genotype	IUGR (n=280) n (%)	Non-IUGR (n=276) n (%)	Allele	IUGR (n=280)	Non-IUGR (n=276)	
*FTO*							
rs1421085 T>C	T/T	111 (39.7)	118 (42.8)	T	0.63	0.65	0.5875
	T/C	132 (47.1)	121 (43.8)	C	0.37	0.35	
	C/C	37 (13.2)	37 (13.4)				
rs17817449 T>G	T/T	134 (47.9)	153 (55.4)	T	0.74	0.78	<0.001
	T/G	146 (52.1)	123 (44.6)	G	0.26	0.22	
rs8043757 A>T	A/A	130 (46.4)	147 (53.3)	A	0.72	0.76	<0.001
	A/T	146 (52.2)	123 (44.6)	T	0.28	0.24	
	T/T	4 (1.4)	6 (2.1)				
rs9926289 G>A	G/G	115 (41.1)	90 (32.6)	G	0.61	0.57	0.9003
	G/A	112 (40.0)	135 (48.9)	A	0.39	0.43	
	A/A	53 (18.9)	51 (18.5)				
rs9939609 T>A	T/T	115 (41.1)	90 (32.6)	T	0.61	0.57	0.9002
	T/A	112 (40.0)	135 (48.9)	A	0.39	0.43	
	A/A	53 (18.9)	51 (18.5)				
*PPARγ*							
rs10865710 C>G	C/C	183 (65.4)	169 (61.2)	C	0.83	0.81	<0.001
	C/G	97 (34.6)	107 (38.8)	G	0.17	0.19	
rs17036263 A>G	A/A	273 (97.5)	269 (97.5)	A	0.99	0.99	1.00
	A/G	7 (2.5)	7 (2.5)	G	0.01	0.01	
rs1801282 C>G	C/C	237 (84.6)	242 (87.7)	C	0.92	0.94	0.6095
	C/G	43 (15.4)	34 (12.3)	G	0.08	0.06	
rs41516544 A>G	A/A	243 (86.8)	273 (98.9)	A	0.93	0.99	1.00
	A/G	37 (13.2)	3 (1.1)	G	0.07	0.01	
rs3856806 C>T	C/C	199 (71.1)	225 (81.5)	C	0.86	0.91	0.1458
	C/T	81 (28.9)	51 (18.5)	T	0.14	0.09	

HWE: hardy Weinberg equilibrium. P-values for each allele and each group are shown.

To decide which SNP to retain, we checked the unadjusted statistical significance of association with IUGR for each SNP separately among males and females (Table 3). Among *FTO* SNPs, we found that both rs9939609 and rs9926289 among males and females had the strongest (P-value=0.059) association with IUGR. Thus, for the analyses of effects of the *FTO* SNPs on IUGR we used only *rs9939609*, which has also been discussed (13). Among the *PPARγ* SNPs, a high degree of collinearity was also observed between certain SNPs. For example, the Fisher exact test for association between the markers rs3856806 and rs41516544 for both males and females was highly significant (P-values <2.2×10^-16^ and 1.102×10^-12^, respectively). Using the same reasoning for choosing an SNP, the only *PPARγ* SNP with a P-value less than 0.001 was rs41516544 and therefore, retained in the study for further analyses.

**Table 3:** Distribution and unadjusted association of genotype and other maternal characteristics with intrauterine growth restriction (IUGR).

Variables^*^	IUGR (n,%)		OR	95%CI	P-value^‡^
	Yes	No			
*FTO* rs9939609					
Male	n=124	n=129			0.059
T/T	54 (43.5)	36 (27.9)	1.00		
T/A	47 (37.1)	69 (53.5)	0.50	0.29-0.79	
A/A	23 (18.5)	24 (18.6)	0.72	0.35-1.49	
Female	n=156	n=147			0.929
T/T	61 (39.1)	54 (36.7)	1.00		
T/A	65 (41.7)	66 (44.9)	0.91	0.55-1.52	
A/A	30 (19.2)	27 (18.4)	1.00	0.53-1.92	
*PPARγ* rs41516544					
Male	n=124	n=129			<0.001
A/A	101 (81.5)	128 (99.2)	1.00		
A/G	23 (18.5)	1 (0.8)	28.29	3.73-214.51	
Female	n=156	n=147			0.011
A/A	142 (91.0)	145 (98.6)	1.00		
A/G	14 (9.0)	2 (1.4)	7.10	1.58-31.98	
Maternal age (years)					
Male					0.543
20-34	97 (78.2)	99 (76.7)	1.00		
≥35	11 (8.9)	16 (12.4)	0.68	0.30-1.55	
<20	16 (12.9)	14 (10.9)	1.22	0.55-2.68	
Female					0.019
20-34	124 (80.0)	114 (77.6)	1.00		
≥35	8 (5.2)	19 (12.9)	0.33	0.13-0.82	
<20	23 (14.8)	14 (9.5)	1.60	0.77-3.31	
Maternal schooling (years)					
Male					0.123
<9	96 (78.7)	86 (68.8)	1.00		
≥9	26 (21.3)	39 (31.2)	0.64	0.36-1.13	
Female					0.685
<9	120 (77.9)	113 (79.6)	1.00		
≥9	34 (22.1)	29 (20.4)	1.12	0.64-1.97	
Maternal smoking					
Male					0.027
No	75 (61.0)	94 (75.2)	1.00		
Yes	48 (39.0)	31 (24.8)	1.86	1.07-3.21	
Female					0.003
No	94 (61.0)	109 (76.8)	1.00		
Yes	60 (39.0)	33 (23.2)	2.15	1.29-3.58	

Variation in number and percentage in some categories of the variables is due to missing information. ^‡^P-value for chi-squared test. *FTO*: fat mass and obesity-associated; *PPARγ*: peroxisome proliferator-activated receptor-gamma.

Table 3 shows the frequency of known IUGR predictors and the selected SNPs as well as their unadjusted associations with IUGR stratified by sex. In this study, the proportion of maternal smoking was higher in IUGR than in non-IUGR participants independent of sex. We also observed that mothers older than 35 years showed a lower rate of IUGR only among female offspring. The rs41516544 SNP from the *PPARγ* gene appears to be a significant risk factor for IUGR independent of sex, while the rs9939609 SNP of the *FTO* gene may have a significant impact on IUGR only for male offspring.

All the variables in Table 3 were included in a multivariate logistic regression to assess their joint effect on IUGR. The three genotypes in the rs9939609 SNP were treated without reference to a specific genetic model such as dominance of one allele or an additive model. For rs41516544, there were only two genotypes present, so the choice of model was less of an issue. All the adjusted ORs along with the 95% confidence intervals are presented in Table 4. As earlier, the results are separated by sex. For both sexes, only individuals with no missing values were retained in the analysis.

**Table 4:** Adjusted associations of *FTO* (fat mass and obesity-associated) and *PPARγ* (peroxisome proliferator-activated receptor-gamma) genotypes and maternal characteristics with intrauterine growth restriction (IUGR).

	Male		Female	
	OR	95%CI	OR	95%CI
*FTO* rs9939609				
TT	1.00		1.00	
TA	0.47	0.26-0.86	1.02	0.60-1.74
AA	0.74	0.34-1.62	1.02	0.51-2.07
*PPARγ* rs41516544				
AA	1.00		1.00	
AG	27.83	3.65-212.32	8.94	1.96-40.88
Maternal age (years)				
20-34	1.00		1.00	
≥35	0.61	0.24-1.56	0.29	0.11-0.83
<20	1.22	0.52-2.88	1.45	0.67-3.12
Maternal schooling (years)				
<9	1.00		1.00	
≥9	0.66	0.36-1.24	1.31	0.72-2.37
Maternal smoking				
No	1.00		1.00	
Yes	1.47	0.81-2.69	1.93	1.14-3.28

Adjusted for maternal age, schooling, and smoking during pregnancy.

We observed that for male offspring the heterozygous AG genotype in the rs41516544 SNP of the *PPARγ* gene was associated with a 28-fold increase in the risk for IUGR, compared with the AA homozygote, while for females there was a 9-fold increase in risk. In male offspring, the heterozygous TA genotype in the rs9939609 SNP of the *FTO* gene appeared to be associated with a lower risk for IUGR compared with the TT genotype. In female offspring, no similar effect was observed. For female offspring, a maternal age of 35 years or more seemed to be associated with a lower risk for IUGR compared with maternal age of 20 to 34 years, which was considered the baseline category. This effect was not observed in male offspring. Finally, maternal smoking was a risk factor only in female offspring, while maternal education did not appear to be a risk factor for children of either sex.

## Discussion

It is generally accepted that the cause for IUGR development is the combination of maternal, placental, fetal, and genetic factors (5,6). In the current study, we conducted an HRM analysis together with DNA sequencing in a Brazilian population from a Ribeirão Preto birth cohort to address genetic risk factors and maternal factors, which may be associated with IUGR.

Our finding of a highly significant association of *PPARγ* rs41516544 on IUGR is the most interesting result in this paper. Other studies have investigated the association between *PPARγ* rs41516544 and type 2 diabetes with increased risk of metabolic complications such as T2DM, insulin resistance, and obesity (18 [Bibr B19],^30^). Thus, finding an SNP that is related to both IUGR and adult metabolic diseases is particularly interesting in light of the association between IUGR and diabetes in adults observed in British populations by Barker et al. (1).

Other SNPs in *PPARγ* have been studied in the context of T2M, in particular rs1801282 (31). The observed size and nature of the association between T2M and rs1801282 are not yet clearly established. In an Indian population, the association is positive but statistically insignificant in non-obese subjects (31). In the same paper, a meta-analysis was carried out using results from many different studies and different ethnic groups. In some of these studies, the OR is significantly larger than 1, and in others, significantly less than 1. Many studies found no evidence for any effect (31). None of the studies cited in that meta-analysis were from populations in Latin America. Furthermore, Morgan et al. (13) found no association between rs1801282 and SGA. In light of these studies on rs1801282 and known associations between IUGR and T2DM, and T2DM with rs41516544, our findings relating rs41516544 with an increased risk for IUGR independent of the sex of the offspring are relevant.

We also investigated potential confounders and environmental factors that might contribute to this association such as maternal smoking, age, and education. Earlier studies have shown that maternal smoking is associated with a reduction in birth weight (32) and this association has been observed in numerous different populations (33,34). In our analysis, we found that maternal smoking increased the risk for IUGR only for female offspring (OR=1.93, 95%CI: 1.14-3.28), suggesting a manifestation of gender differences on the effects of cigarette smoking. In fact, Lockhart et al. observed a significant increase of serotonin in smoking mothers carrying male fetuses compared with smoking mothers carrying female fetuses (35). However, factors associated with the intrauterine environment, which lead to these differences, require further research.

This is the first study to report data on the prevalence of alleles of *FTO* and *PPARγ* genes associated with IUGR in Brazil. However, we were unable to include maternal variables that are related to the intrauterine growth of the offspring, such as maternal pre-pregnancy weight and weight gain during gestation (36), as they were not available in the original birth cohort. Nonetheless, the environmental factors and potential confounders included do appear to play a role in IUGR.

Maternal age appears to be associated with a reduced risk for IUGR female offspring only and for mothers above the age of 35 only (OR=0.29, 95%CI: 0.11-0.83), also suggesting a gender-specific response whose mechanism remains unknown. The association between maternal age and risk for IUGR from other studies is ambiguous. A population-based study from a Northeastern Brazilian city, where IUGR rates of 16.2% are observed, has shown no significant association between maternal age and increased prevalence of IUGR (37). However, in another meta-analysis including 63 cohort studies and 12 case-control studies, higher maternal age increases the risk of fetal growth restriction (38). Furthermore, the design of most studies does not consider male and female offspring separately as we did in the current study, and therefore the mechanisms of these maternal age effects require further research that must consider both genders separately and include genetic, epigenetic, demographic, and socioeconomic factors.

In our study, we also demonstrated that *FTO* rs9939609 TA genotype was associated with a reduced risk for IUGR in male offspring only (OR=0.47, 95%CI: 0.26-0.86). The *FTO* rs9939609 variant is a well-studied SNP that has been associated with higher BMI, risk of obesity, and subsequent T2DM development (15,16,20). A meta-analyses conducted by Peng et al. (21) demonstrated the correlation between *FTO* rs9939609 and obesity, which was observed in 21 of the 29 studies. These data have shown that the association of *FTO* rs9939609 and risk for obesity might depend on ethnicity. Furthermore, the studies analyzed in Peng et. al. (21) included only one South American population from Mexico. Hence, additional studies in different ethnic groups are important to get a clearer picture of the effects of rr993609. Our data show that the *FTO* rs9939609 TA genotype has a protective effect against IUGR, but only in male offspring with no effect in female offspring, suggesting that gender-specific factors may underlie these differences. Although the understanding of the full details of the relationship between *FTO* with IUGR is not complete, the results shown here are of interest.

In conclusion, we found a gender-specific association of the *FTO* rs9939609 TA genotype with IUGR, showing a reduced risk among male offspring. On the other hand, the *PPARγ* rs41516544 AG genotype showed a highly significant risk of IUGR in both sexes. These associations were found even after controlling for non-genetic confounding factors. These results are interesting, but should be validated in a larger study in an independent birth cohort.
